# Elevated SerpinB2 regulates MUC5AC expression via STAT6 signaling in nasal epithelial cells in allergic rhinitis

**DOI:** 10.3389/fimmu.2025.1669777

**Published:** 2025-10-10

**Authors:** Jing Li, Qianbo Cui, Zhipeng Li, Ying Zhu, Jing Wang, Wei Chen

**Affiliations:** ^1^ Department of Otorhinolaryngology Head and Neck Surgery, The Central Hospital of Wuhan, Tongji Medical College, Huazhong University of Science and Technology, Hubei, China; ^2^ Department of Otolaryngology Head and Neck Surgery, Shanghai Sixth People’s Hospital Affiliated to Shanghai Jiao Tong University School of Medicine, Shanghai, China; ^3^ Department of Otorhinolaryngology Head and Neck Surgery, Tongji Hospital, Tongji Medical College, Huazhong University of Science and Technology, Hubei, China

**Keywords:** SerpinB2, MUC5AC, epithelial cell, allergic rhinitis, STAT6

## Abstract

**Background:**

SerpinB2 is expressed in airway epithelial cells in nasal polyps and asthma in association with Type-2 inflammation. Allergic rhinitis (AR) is similarly associated with Type-2 inflammation and mucus hypersecretion. MUC5AC has been reported to be regulated by IL-13 in nasal epithelial cells (NECs). This study aimed to evaluate SerpinB2 expression in AR and determine whether SerpinB2 regulates MUC5AC via STAT6 signaling.

**Methods:**

SerpinB2 gene expression in single-cell RNA sequencing databases was analyzed through bioinformatics approaches. SerpinB2 and MUC5AC expression levels were evaluated in intermittent or persistent AR patients and healthy controls (HC). Colocalization of SerpinB2 and MUC5AC was assessed by immunofluorescence. Fresh NECs were cultured at air-liquid interface with or without IL-13, SerpinB2 Dicer-substrate short interfering RNAs (DsiRNAs) transfection, exogenous SerpinB2, and pSTAT6 inhibitors. SerpinB2, MUC5AC, and STAT6 were analyzed using qRT-PCR, Western blot, immunofluorescence, and ELISA.

**Results:**

SerpinB2 expression was significantly increased in both intermittent and persistent AR compared to normal mucosa from HC. SerpinB2 correlated with MUC5AC expression and colocalized with MUC5AC in NECs from AR patients. In primary NECs *in vitro*, IL-13 induced both SerpinB2 and MUC5AC expression. Knockdown or overexpression of SerpinB2 correspondingly decreased or increased MUC5AC expression in NECs. STAT6 inhibition similarly reduced SerpinB2-induced MUC5AC expression.

**Conclusions:**

SerpinB2 is upregulated in AR NECs and contributes to MUC5AC expression through STAT6 signaling pathway activation. Therefore, targeting SerpinB2 may have therapeutic value in treating AR patients.

## Introduction

Allergic rhinitis (AR) is a chronic inflammation of the nasal mucosa driven by IgE mediated hypersensitivity reaction to allergens (1–3). Mucus hypersecretion is a remarkable characteristic of AR that contributes to nasal obstruction, rhinorrhea and other symptoms (4). MUC5AC, a major gel-forming mucin, is predominantly expressed in goblet cells of the respiratory tract and is upregulated in allergic diseases (5). In AR, exposure to allergens leads to a cascade of immune responses, including the release of Type-2(T2) cytokines like IL-4, IL-5, and IL-13. IL-13 induces goblet cell hyperplasia and upregulating MUC5AC transcription through the activation of the STAT6 signaling pathway (6). Excessive MUC5AC production increases mucus viscosity and volume, causes impaired mucociliary clearance, nasal congestion, and prolonged inflammation (7). Understanding the regulation of MUC5AC by T2 cytokines provides valuable insights into the pathophysiology of AR and highlights the need for targeted therapies that address mucus hypersecretion.

SerpinB2, also known as plasminogen activator inhibitor type 2 (PAI-2), is a member of the serine protease inhibitor superfamily and is primarily expressed in airway epithelial cells (8). Studies have shown that SerpinB2 is significantly elevated in the bronchial epithelial cells of asthmatic patients and is associated with pivotal clinical markers, such as FEV1, FeNO, eosinophil counts and asthma severity (9–11). Single-cell sequencing analyses have identified SerpinB2 as one of the core upregulated genes in human bronchial epithelial cells after exposure to IL-13 (12). Additionally, *in vitro* studies have showed that IL-13 induce SerpinB2 expression in primary bronchial epithelial cells, which can be reversed by corticosteroids (13). In our previous study, we found that SerpinB2 induced by IL-13 contributes to the expression of 15-lipoxygenase-1 (15LO1) and its downstream markers, such as CCL26 and iNOS in nasal polyp epithelial cells (14). Based on these findings, we hypothesized that elevated levels of SerpinB2 in AR could increase the expression of MUC5AC, thereby contributing to mucus hypersecretion in AR.

To test this hypothesis, fresh nasal epithelial cells (NECs) from intermittent AR (iAR), persistent AR (pAR) patients and healthy controls (HC) were analyzed for the expression of SerpinB2, MUC5AC, MUC5B, CCL24, CCL26, ALOX15 and POSTN. Correlation analyses were conducted to assess the relationship between SerpinB2 and other genes. Additionally, NECs were cultured under air-liquid interface (ALI) conditions to investigate the regulatory role of SerpinB2 on MUC5AC expression and the underlying molecular mechanisms. Our results demonstrate that SerpinB2 is upregulated in NECs from iAR and pAR patients and plays a regulatory role in MUC5AC production. These findings provide new insights into the pathophysiology of AR and suggest that SerpinB2 may be a key mediator of mucus dysfunction in AR.

## Methods

### Study participants

A total of 15 control subjects, 16 patients with iAR, and 14 patients with pAR were included in this study. The inferior turbinate (IT) were collected during the septal plastic surgery. Given the limited tissue quantity, not all tissue samples were included in every experimental protocol. The diagnostic criteria of AR were referred to the Chinese Guideline for Diagnosis and Treatment of Allergic Rhinitis (2022, revision) (1). Atopy to HDM was determined by a positive skin prick test response to Dermatophagoides pteronyssinus (Der p) and/or Dermatophagoides farinae (Der f), or serum specific IgE to Der p and/or Der f ≥ 0.776 KU/L (1). AR patients who were sensitized to inhalant allergens other than HDM, such as pollens and fungi were excluded. Subjects with chronic rhinosinusitis, previous treatment with immunotherapy, severe immunologic, chronic infection or acute infection in a month were excluded from this study. Antihistamines or intranasal steroid sprays were discontinued for at least 2 weeks, and oral steroids were discontinued at least 3 months before inclusion. This study was approved by the Ethics Committee of The Central Hospital of Wuhan, and written informed consent was obtained from all patients. Nasal secretions were collected from middle meatus bilaterally with scissored sponge pack Merocel as previously reported. The number of nasal secretion samples did not fully match the number of tissue samples because not all participants provided both specimen types. In addition, some nasal secretion samples yielded insufficient volume or failed to meet quality criteria for ELISA analysis, and were therefore excluded. For details, see the Online Repository.

### Bioinformatic analysis of the scRNA-seq datasets of allergic rhinitis

Single-cell RNA-sequencing (scRNA-seq) data from the Gene Expression Omnibus (GEO) database (accession number GSE261706) were reanalyzed using standard bioinformatics protocols (15). The dataset comprised IT samples collected from two control subjects and two patients with allergic rhinitis (AR). Detailed methodological procedures are provided in the Online Repository.

### Immunofluorescent staining and confocal microscopy

For IF staining, tissues sections and cells were incubated with primary antibodies against SerpinB2 and MUC5AC. Sections and cells were then incubated with Alexa 488 and 594 conjugated secondary antibodies, counterstained with DAPI and imaged using Zeiss confocal laser scanning microscope (LSM 780). For details, see the online supplement.

### Quantitative real-time PCR

qRT-PCR was performed using a SYBR method and Roche LightCycler 480 II System. *GUSB* was used for internal control. Relative mRNA expression levels were calculated using the ΔCt method. For details, see the online supplement.

### Western blot

Fresh nasal tissue samples and cell lysates were run on SDS-PAGE gels as previously described. Western blot was performed using primary antibodies against SerpinB2, GAPDH, pSTAT6, and tSTAT6. Densitometry analysis was performed with ImageJ software. For details, see the online supplement.

### Primary HNECs culture at the ALI

NECs were obtained from HC subjects by epithelial brushings of the IT. Primary NECs were cultured in ALI as previously described. For details, see the online supplement.

### Short interfering RNA transfection of primary HNECs under ALI stage

*SerpinB2* siRNA transfection was performed using Lipofectamine RNAiMAX reagent. After 24 hours, the transfection mixture was removed and switched to ALI culture. Cells were stimulated with or without IL-13 under ALI culture. For details, see the online supplement.

### MUC5AC ELISA

MUC5AC protein levels in the nasal secretion and cell supernatants were measured with a quantitative sandwich ELISA, according to the manufacturer’s instructions.

### Statistical analysis

The bioinformatics analysis and graphic production were performed using R version 4.3.1 (Foundation for Statistical Computing). For the experimental data, all statistical analysis was performed with JMP Pro software (SAS Institute, Cary, NC). Graphic productions were performed using GraphPad Prism 9 (GraphPad Software). Normally distributed data were represented as means ± SEMs, nonnormally distributed data were represented as medians with a 25% to 75% interquartile range. Correlation analyses were performed by using Spearman rank correlation. P values of less than 0.05 were considered statistically significant.

## Results

### Demographics

Fresh NECs were obtained from a total of 15 HCs and 30 AR subjects. Of the 30 AR subjects, 16 were defined as iAR and 14 were defined as pAR (**Table 1**). For the 15 HCs, all NECs were collected from IT. Due to the limited cell numbers, not all cells were used for all experiments.

**Table 1:** Clinical characteristics of subjects.

All subjects^1^	HC	iAR	pAR	P value
N	15	16	14	
Age (mean ± SD)	31.32 ± 8.04	39.15 ± 13.07	38.87 ± 15.25	0.162
Sex (Male/Female)	11/4	13/3	11/3	0.138
Asthma (Y/N)	0/15	1/15	1/13	>0.999
PBE counts (mean ± SD)^2^	0.04 ± 0.08	0.24 ± 0.16	0.21 ± 0.19	0.001
PBE % (mean ± SD)^3^	1.34 ± 1.14	3.14 ± 2.31	2.94 ± 1.48	0.012
Specific IgE(mean ± SD)^4^	24.51 ± 17.59	154.71 ± 38.05	178.47 ± 58.38	<0.0001

^1^HC, health control; iAR, intermittent allergic rhinitis; pAR, persistent allergic rhinitis.

^2^PBE counts, peripheral blood eosinophil counts (×10^9^/L).

^3^PBE %, PBE percentage.

^4^Specific IgE: KU/L.

### Bioinformatics analysis revealed SerpinB2 expression was markedly upregulated in epithelial cells from AR patients

**Figure 1:**
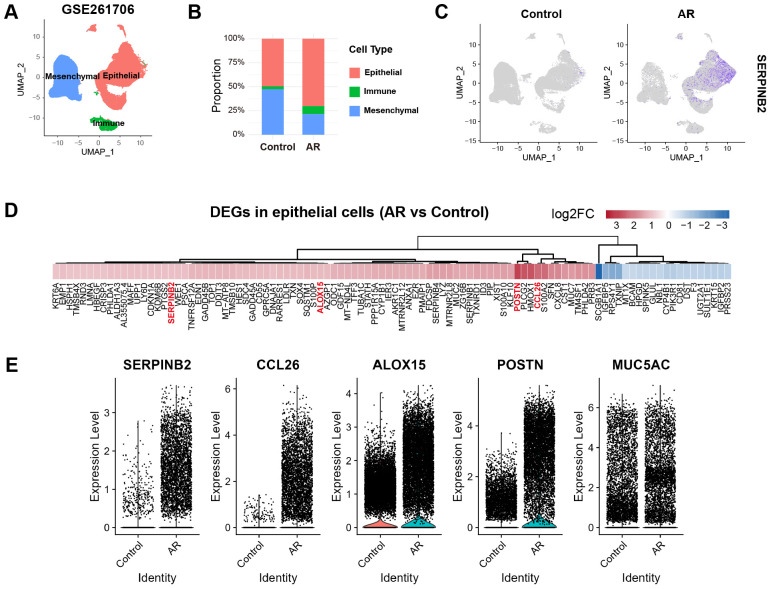
Reanalysis of scRNA-seq data reveals increased *SerpinB2* expression in epithelial cells from AR patients. **(A)** UMAP visualization of cells from 2 control and 2 AR subjects (GSE261706) clustered into 3 major cell types. **(B)** Percentage of epithelial cells, immune cells and mesenchymal cells in control and AR groups. **(C)** Feature plots displaying the cellular distribution of *SerpinB2* expression in control and AR groups. **(D)** Heatmap of the top 100 differentially expressed genes (DEGs) in epithelial cells from AR group compared to the control group, ranked by |log2FC|. **(E)** Relative expression levels of *SerpinB2, CCL26, ALOX15, POSTN* and *MUC5AC* in epithelial cells from control and AR groups.

To evaluate *SerpinB2* expression in epithelial cells, we analyzed previously published scRNA-seq data of nasal mucosa obtained from control subjects and AR patients. Following preprocessing and quality control, we identified 3 major cell types based on established marker genes: epithelial cells (*EPCAM, KRT8, KRT5*), mesenchymal cells (*COL1A1, ACTA2, PDGFRA*), and immune cells (*PTPRC, CD3E, MS4A1, HLA-DRA*) (**Figure 1A**). The proportions of epithelial cells and immune cells were markedly increased in AR group compared to control group (**Figure 1B**). *SerpinB2* was predominantly expressed by epithelial cells and showed significantly elevated expression in the AR group (**Figure 1C**). To further investigate this finding, we performed differential gene expression analysis comparing epithelial cells between AR and control groups. *SerpinB2* expression was significantly upregulated in AR epithelial cells, along with several genes associated with type 2 immunity, including *CCL26, ALOX15*, and *POSTN* (**Figures 1D, E**).

### Increased expression of SerpinB2 in AR NECs and associated with MUC5AC expression

**Figure 2:**
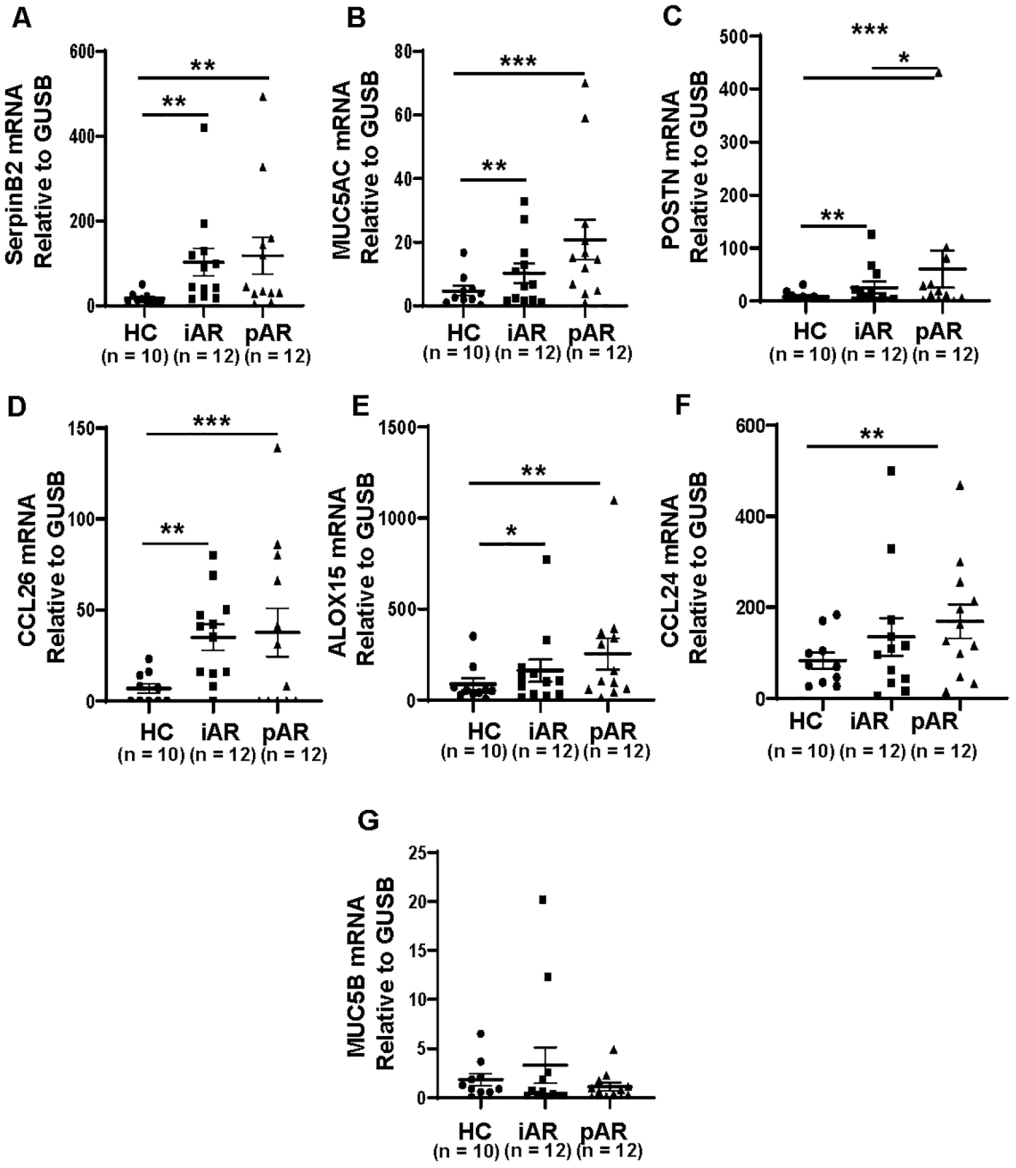
Increased expression of *SerpinB2, MUC5AC, CCL26, ALOX15* and *POSTN* in iAR and pAR NECs compared with HC. **(A)** The mRNA expression levels of *SerpinB2* in NECs of patients (n=34) in different study groups by means of qRT-PCR. **(B)** The mRNA expression levels of *MUC5AC* in NECs of patients (n=34) in different study groups by means of qRT-PCR. **(C)** The mRNA expression levels of *POSTN* in NECs of patients (n=34) in different study groups by means of qRT-PCR. **(D)** The mRNA expression levels of *CCL26* in NECs of patients (n=34) in different study groups by means of qRT-PCR. **(E)** The mRNA expression levels of *ALOX15* in NECs of patients (n=34) in different study groups by means of qRT-PCR. **(F)** The mRNA expression levels of *CCL24* in NECs of patients (n=34) in different study groups by means of qRT-PCR. **(G)** The mRNA expression levels of *MUC5B* in NECs of patients (n=34) in different study groups by means of qRT-PCR. *P < 0.05, **P < 0.01, ***P < 0.001.

To verify whether SerpinB2 is differentially expressed in NECs from AR and HC, *SerpinB2, MUC5AC, MUC5B, CCL24, CCL26*, *ALOX15* and *POSTN* mRNA levels were analyzed in fresh NECs from patients with iAR, pAR and HCs by qRT-PCR. *SerpinB2*, *MUC5AC CCL26*, *ALOX15* and *POSTN* mRNA expression were significantly increased in both iAR and pAR patients, as compared with HC (**Figures 2A–E**), which are consistent with the above bioinformatic results. *CCL24* mRNA expression was increased in pAR tissues, but not in iAR tissues compared with HC (**Figure 2F**). The expression of *MUC5B* mRNA were not statistically different between iAR, pAR and HCs (**Figure 2G**).

**Figure 3:**
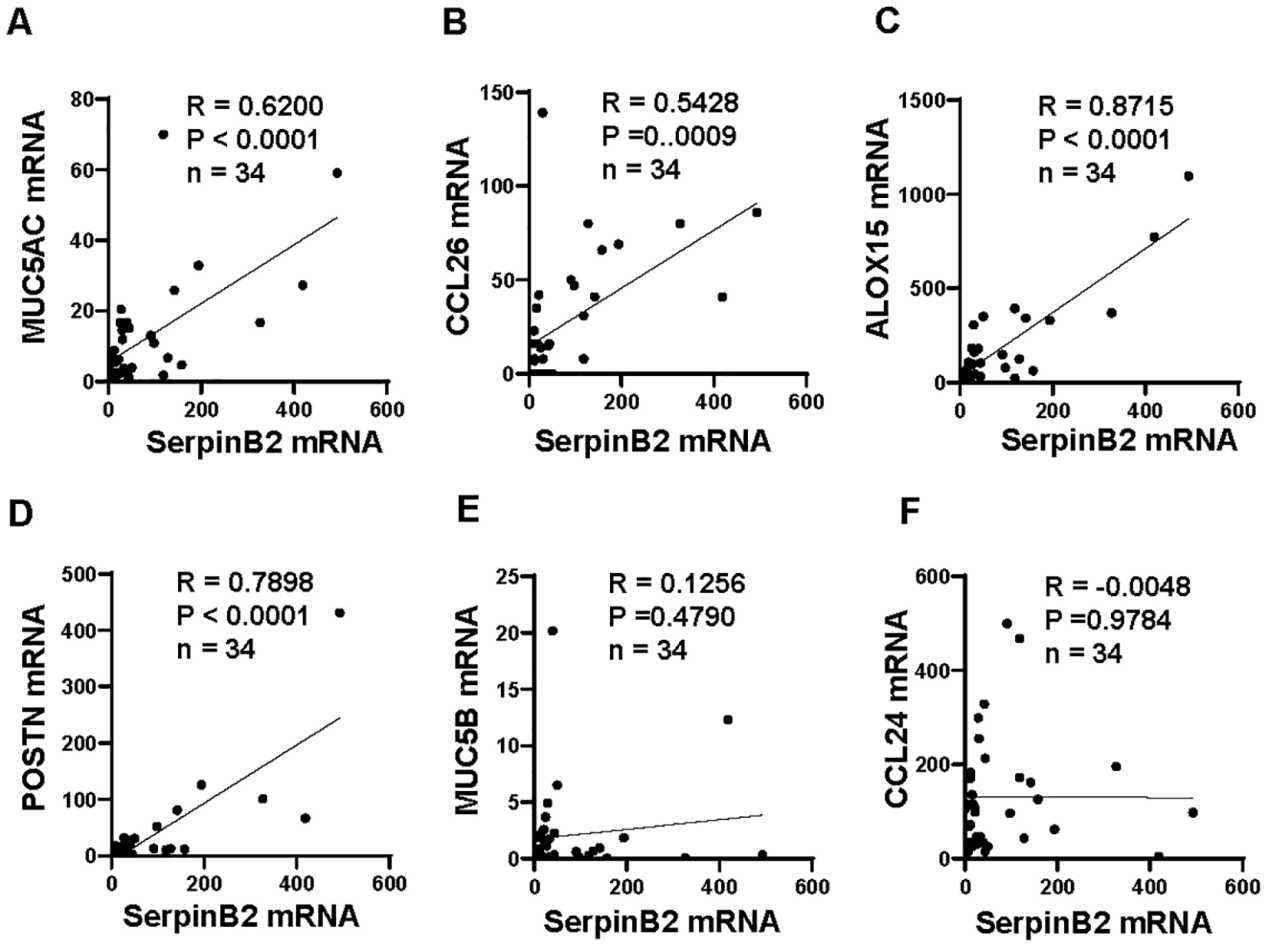
*SerpinB2* mRNA expression correlated with *MUC5AC, CCL26, ALOX15* and *POSTN* mRNA expression *ex vivo*. **(A)** *SerpinB2* mRNA expression positive correlated with *MUC5AC* mRNA expression (n=34). **(B)** *SerpinB2* mRNA expression positive correlated with *CCL26* mRNA expression (n=34). **(C)** *SerpinB2* mRNA expression positive correlated with *ALOX15* mRNA expression (n=34). **(D)** *SerpinB2* mRNA expression positive correlated with *POSTN* mRNA expression (n=34). **(E)** *SerpinB2* mRNA expression was not correlated with *MUC5B* mRNA expression (n=34). **(F)** *SerpinB2* mRNA expression was not correlated with *CCL24* mRNA expression (n=34). *P < 0.05, **P < 0.01, ***P < 0.001.

To determine whether *SerpinB2* mRNA expression was related to *MUC5AC, MUC5B, CCL24, CCL26, ALOX15* and *POSTN* mRNA expression. The mRNA correlations were analyzed according to statistical principles. The *SerpinB2* expression was significantly positive correlated with *MUC5AC* expression (r=0.6200, p<0.0001, **Figure 3A**), *CCL26* expression (r=0.5428, p=0.0009, **Figure 3B**), *ALOX15* expression (r=0.8715, p<0.0001, **Figure 3C**) and *POSTN* expression (r=0.7898, p<0.0001, **Figure 3D**), but not with *MUC5B* expression (r=0.1256, p=0.4790, **Figure 3E**) and *CCL24* expression (r=-0.0048, p=0.9784, **Figure 3F**).

**Figure 4:**
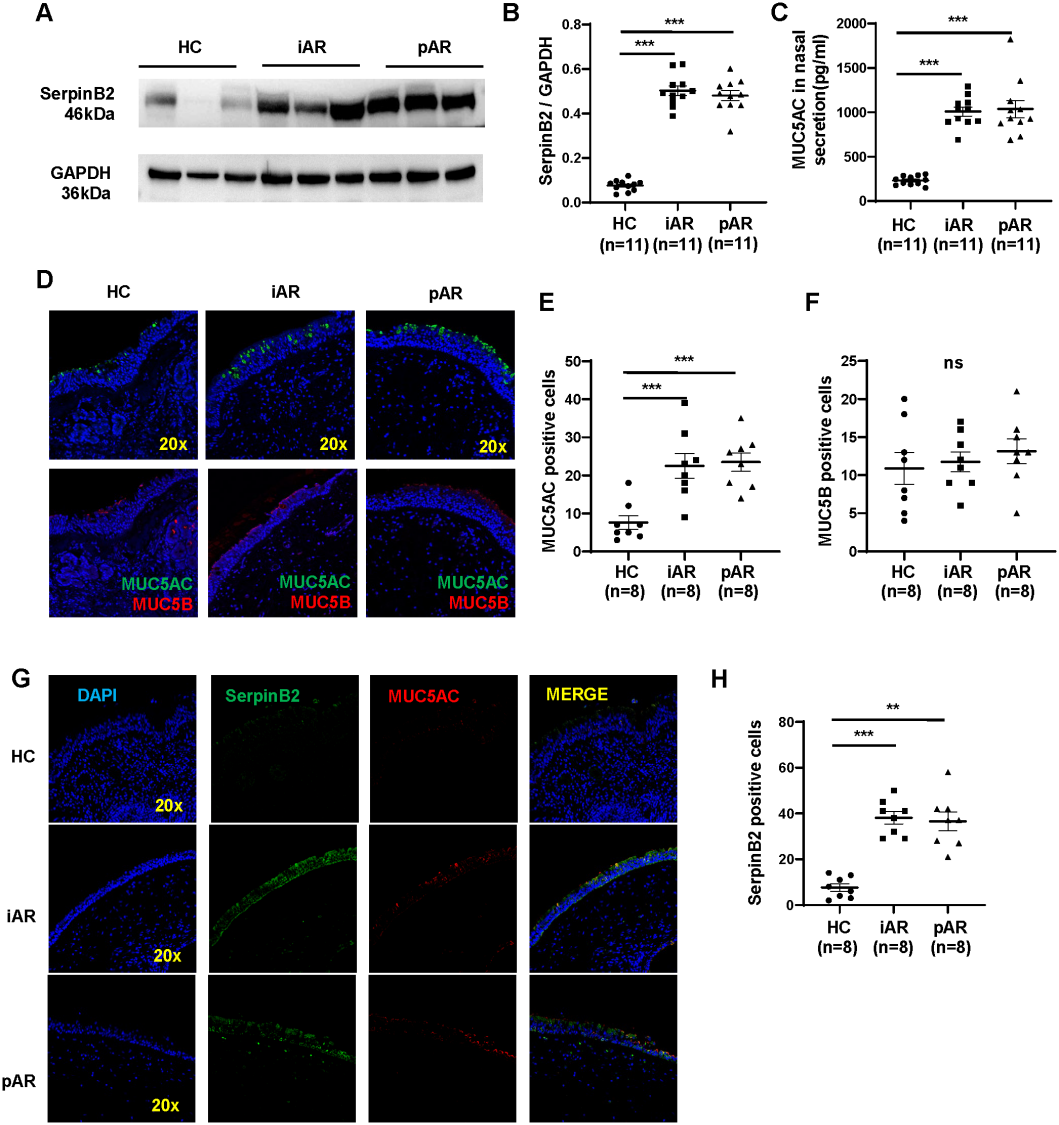
Increased expression of SerpinB2 and MUC5AC protein in iAR and pAR NECs compared with HC. **(A)** The protein expression levels of SerpinB2 in NECs of patients (n=33) in different study groups by means of Western blotting. **(B)** Densitometric analysis of SerpinB2 protein from different groups by using WB. **(C)** The protein expression levels of MUC5AC in nasal secretion of patients (n=33) in different study groups by means of ELISA. **(D)** Representative immunostaining graph showing that increased MUC5AC in epithelial cells of patients with AR (magnification 20×). **(E)** Quantitation of MUC5AC positive cells in nasal tissues from AR and HC. **(F)** Quantitation of MUC5B positive cells in nasal tissues from AR and HC. **(G)** Representative double immunofluorescence staining showing SerpinB2 (green) and MUC5AC (red) in nasal tissues from AR and HC (magnification 20×). **(H)** Quantitation of SerpinB2 positive cells in nasal tissues from AR and HC. **P < 0.01, ***P < 0.001. ns, not significant.

To further determine whether SerpinB2 and MUC5AC protein is differentially expressed in NECs from iAR, pAR and HC, SerpinB2 and MUC5AC protein levels were analyzed in freshly isolated NECs and nasal secretions by WB and ELISA. SerpinB2 expression at the protein level was similarly upregulated in both iAR and pAR tissues (**Figures 4A, B**). MUC5AC protein expression in the nasal secretion were significantly higher in iAR and pAR than in control tissues (**Figure 4C**).

To determine the colocalization of SerpinB2 and MUC5AC in AR nasal tissues, immunofluorescence staining was performed. Consistent with previous qRT-PCR and WB results, more MUC5AC-positive epithelial cells were observed in NECs from iAR and pAR patients compared with HC, whereas the number of MUC5B-positive cells showed no significant difference (**Figures 4D–F**). Confocal co-immunostaining further demonstrated increased SerpinB2 expression in epithelial cells, with clear colocalization of SerpinB2 and MUC5AC in AR tissues (**Figures 4G, H**).

### IL-13 induces SerpinB2 and MUC5AC expression *in vitro*

**Figure 5:**
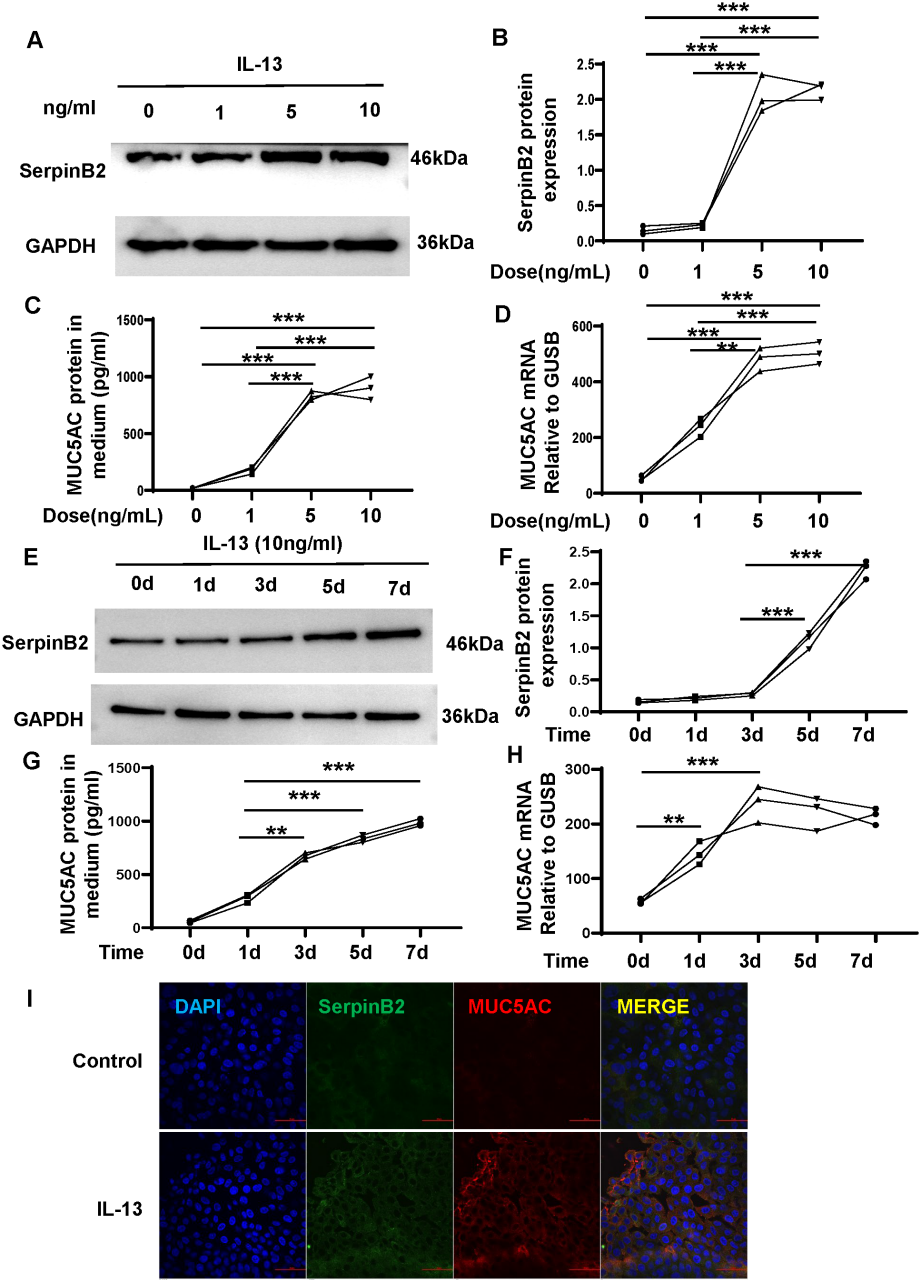
IL-13 induces SerpinB2 and MUC5AC expression in ALI cultured NECs *in vitro*. **(A)** SerpinB2 protein expression of NECs treated with different concentrations of IL-13 treatment by means of WB. **(B)** Densitometric analysis of SerpinB2 protein expression levels (n=3). **(C)** MUC5AC protein expression of supernatants treated with different concentrations of IL-13 treatment by means of ELISA. (n=3). **(D)** MUC5AC mRNA expression levels of NECs with different concentrations of IL-13 by means of qRT-PCR. **(E)** SerpinB2 protein expression levels of NECs with 10ng/mL of IL-13 treatment at different times by means of WB (n=3). **(F)** Densitometric analysis of SerpinB2 protein expression levels (n=3). **(G)** MUC5AC protein expression of supernatants treated with 10ng/mL of IL-13 treatment at different times by means of ELISA. (n=3) **(H)** MUC5AC mRNA expression levels of NECs with IL-13 at different times by means of qRT-PCR. **(I)** Immunostaining results showed that IL-13 increased SerpinB2/MUC5AC positive cell numbers in ALI cultured NECs (n=3). *P < 0.05, **P < 0.01, ***P < 0.001.

To determine whether SerpinB2 and MUC5AC in NECs respond to IL-13 in a similar manner as nasal polyps, ALI cultured NECs were treated with different concentrations of IL-13 (0 to 10 ng/mL) and for 5 days (14). All data for cell experiments are representative of at least three independent biological experiments. IL-13 increased SerpinB2 protein in cell lysates in a dose dependent manner (**Figures 5A, B**). Similarly, IL-13 induced MUC5AC protein in supernatants and mRNA in cell lysates in a dose dependent manner (**Figures 5C, D**). Then NECs were treated with IL-13(10 ng/mL) for 0 to 7 days (14). SerpinB2 protein increased on Day 3 and were generally sustained for 4 days (**Figure 5E**). IL-13 time dependently increased SerpinB2 protein in cell lysates (**Figure 5F**) and MUC5AC expression in supernatants and cell lysates (**Figures 5G, H**). Thus, 10ng/mL IL-13 for 5 days was used in further studies and comparisons. The immunofluorescent staining showed 10ng/mL IL-13 for 5 days significantly increased SerpinB2/MUC5AC positive staining in cultured NECs (**Figure 5I**).

### SerpinB2 siRNA downregulates IL-13-induced MUC5AC expression in NECs

**Figure 6:**
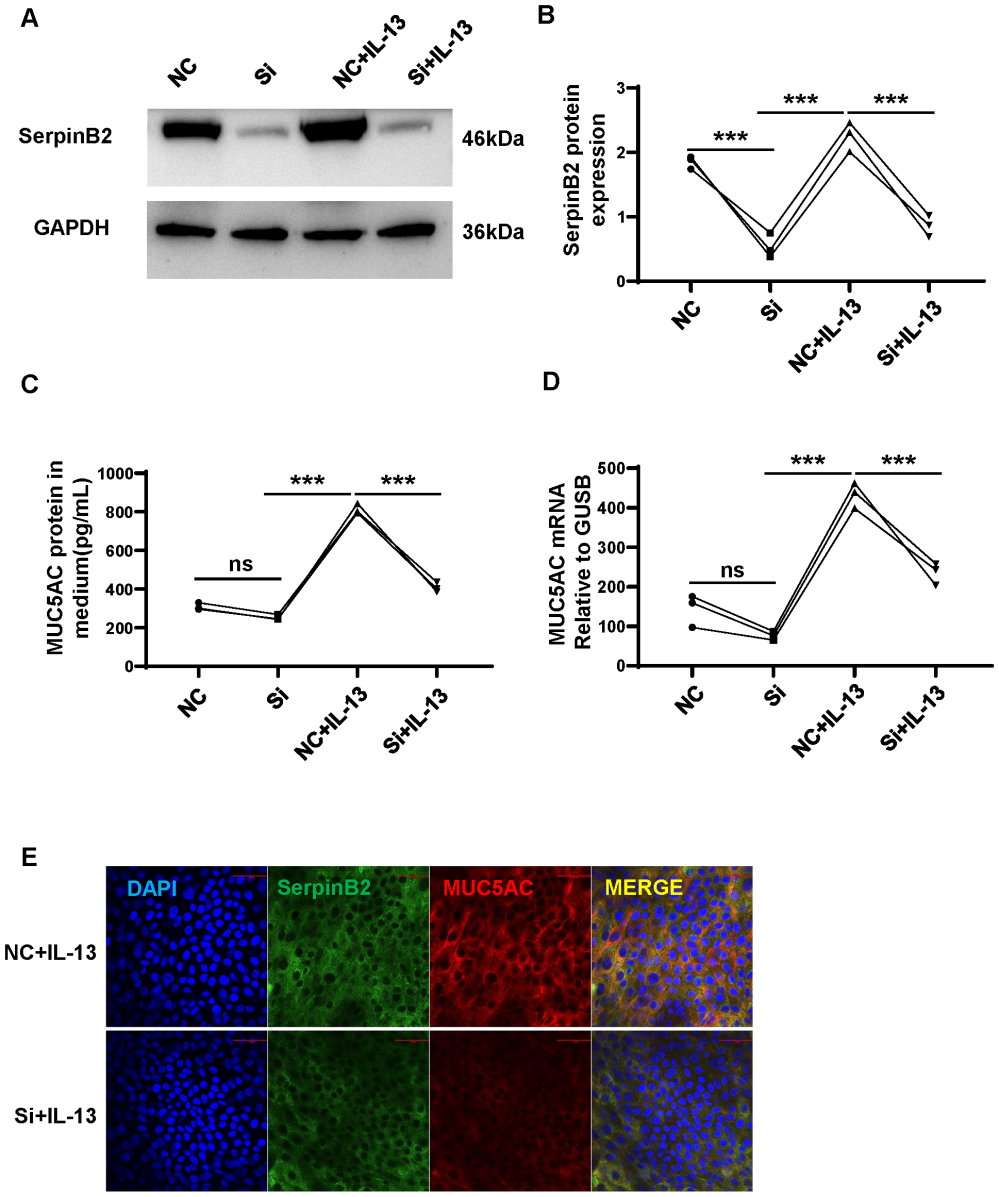
SerpinB2 siRNA downregulates IL-13 induced MUC5AC expression. **(A)** SerpinB2 siRNA decreases intracellular SerpinB2 protein expression (n=3). **(B)** Densitometry analysis of intracellular SerpinB2 protein in cell lysates (n=3). **(C)** SerpinB2 siRNA decreases MUC5AC protein expression in supernatants by using ELISA (n=3). **(D)** SerpinB2 siRNA decreases intracellular MUC5AC mRNA expression by using qRT-PCR (n=3). **(E)** Immunostaining results showed that SerpinB2 siRNA downregulates IL-13 induced MUC5AC in ALI cultured NECs (n=3). ***P < 0.001. ns, not significant.

Previous results showed that SerpinB2 positive correlated with MUC5AC expression in AR. To determine whether SerpinB2 regulated MUC5AC production in NECs, *SerpinB2* silencing was performed with Dicer siRNA transfection in ALI cultured NECs in the absence and presence of IL-13. *SerpinB2* siRNA treatment inhibited IL-13 induced MUC5AC production in epithelial cells at both the mRNA and protein levels (**Figures 6A–D**). The immunofluorescent staining showed *SerpinB2* siRNA significantly decreased SerpinB2/MUC5AC positive staining in cultured NECs (**Figure 6E**).

### Exogenous SerpinB2 increases MUC5AC expression in NECs

**Figure 7:**
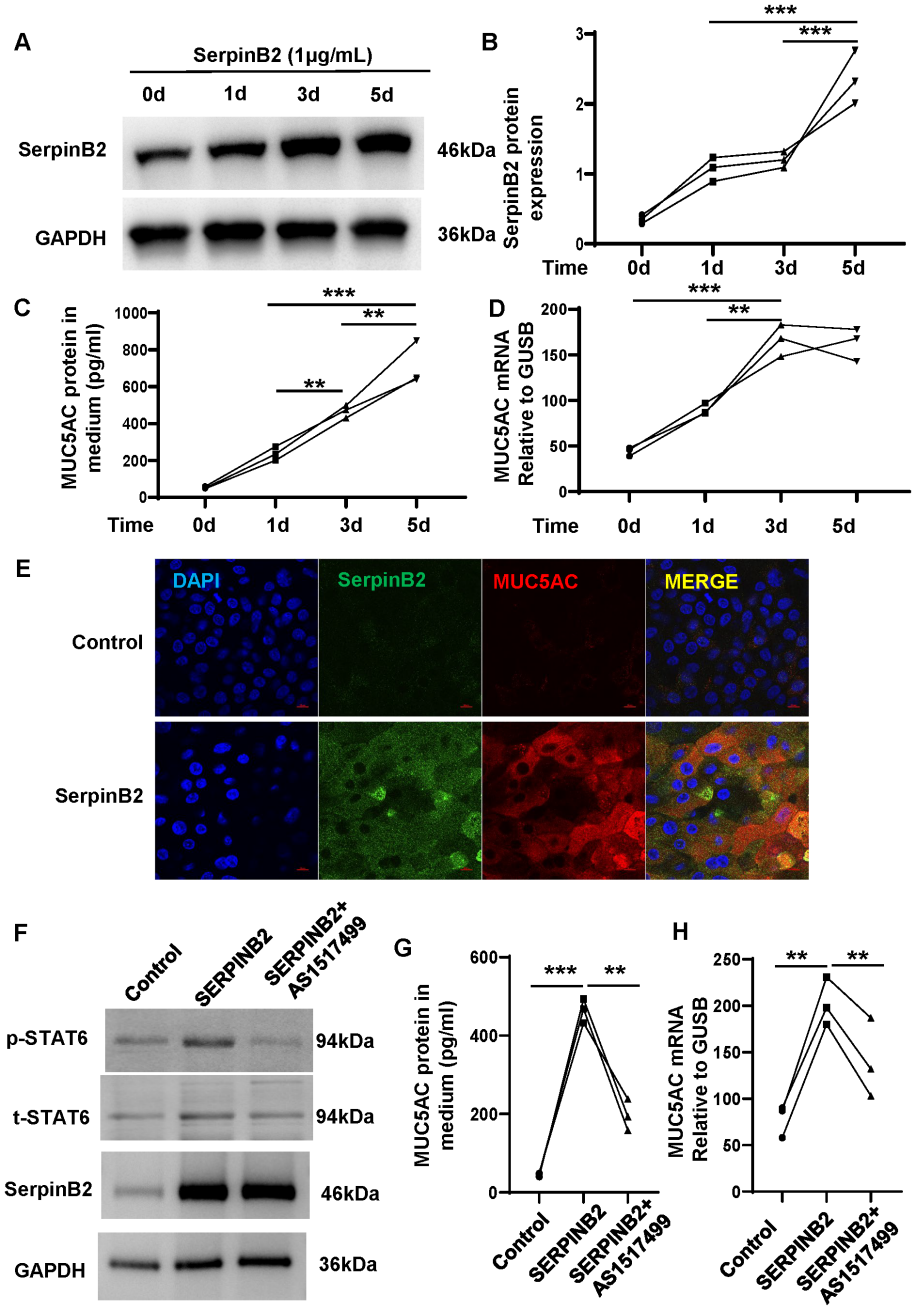
Exogenous SerpinB2 increases MUC5AC expression by activating STAT6 signaling pathway. **(A)** Exogenous SerpinB2 increases intracellular SerpinB2 protein expression (n=3). **(B)** Densitometry analysis of intracellular SerpinB2 protein in cell lysates (n=3). **(C)** Exogenous SerpinB2 increases MUC5AC protein expression in supernatants by using ELISA (n=3). **(D)** Exogenous SerpinB2 increases intracellular MUC5AC mRNA expression by using qRT-PCR (n=3). **(E)** Immunostaining results showed that Exogenous SerpinB2 upregulates IL-13 induced MUC5AC in ALI cultured NECs (n=3). **(F)** pSTAT6 and tSTAT6 protein expression levels of NECs treated with recombinant SERPINB2 protein in the presence or absence of AS1517499(100nM) by means of WB (n=3). **(G)** AS1517499 decreases SerpinB2 induced MUC5AC protein expression in supernatants by using ELISA (n=3). **(H)** AS1517499 decreases SerpinB2 induced MUC5AC mRNA expression by using qRT-PCR (n=3). **P < 0.01, ***P < 0.001.

To confirm the effects of SerpinB2 on MUC5AC expression, exogenous recombinant human SerpinB2 protein treatment was performed. Exogenous SerpinB2 increased MUC5AC gene and protein expression in a time-dependent manner (**Figures 7A–D**). The immunofluorescent staining showed Exogenous SerpinB2 increased SerpinB2/MUC5AC positive staining in cultured NECs (**Figure 7E**).

### SerpinB2 regulate MUC5AC expression by STAT6 signaling

Our previous studies have demonstrated that SerpinB2 induces 15LO1 expression by activating STAT6 signaling. To determine whether SerpinB2 promote MUC5AC expression by activating STAT6 pathway, ALI cultured primary NECs were treated with exogenous recombinant human SerpinB2 and phaspho-STAT6 inhibitor AS1517499 for 3 days. Exogenous recombinant human SerpinB2 enhanced STAT6 phosphorylation and MUC5AC expression while AS1517499 decreased STAT6 phosphorylation and MUC5AC expression level. These results suggest that SerpinB2 partly potentiates MUC5AC expression via STAT6 signaling in epithelial cells (**Figures 7F–H**).

## Discussion

AR has been one of the most prevalent upper airway diseases in the world, affecting 10–30% of the global population and leading to a significant reduction in quality of life and social functioning (16–18). Unfortunately, this disease remains incurable, and the treatment has long been “symptom-control” oriented, which requires long-term medication and results in a series of adverse reactions (19, 20). Therefore, identification of novel biomarkers and therapeutic targets for the treatment of AR is urgent and essential. In the present study, with the inclusion of datasets of epithelial samples and nasal secretion samples, we first demonstrated that SerpinB2 expression levels were clearly elevated in AR patients, include iAR and pAR patients. Moreover, SerpinB2 expressions were strongly correlated with the degree of MUC5AC expression and tissue eosinophilic inflammation. Given that, our findings hinted that SerpinB2 might be involved in the pathogenesis of AR and contribute to mucus hypersecretion and tissue eosinophilic inflammation. Thereby, SerpinB2 appeared to be a novel indicator for the development of therapeutic strategies for AR.

Recent studies demonstrated SerpinB2 could modulate allergic airway inflammation disease inflammatory responses as well as asthma and nasal polyps. (8, 9, 11, 14) However, data supporting the potential role of SerpinB2 in AR are limited, with a previous study reporting aryl hydrocarbon receptor (AhR) promote inflammatory cytokines and chemokines release and epithelial barrier disruption in AR via upregulating SerpinB2 (21). In the current study, In the present study, we analyzed the the cellular distribution of *SerpinB2* among AR and HC groups by using single-cell RNA sequencing dataset. The *SerpinB2* gene was seen at persistently high levels in epithelial cells but not in immune cells and mesenchymal cells. Further we showed that SerpinB2 was significantly increased in the NECs from both iAR and pAR patients. SerpinB2 was demonstrated to be upregulated in bronchial epithelial cells and could promote the T2 inflammatory response in asthma patients and mouse models. (22, 23) A recent single-cell RNA sequencing study of asthma showed that SerpinB2 is one of the core genes which upregulated in all subtypes of bronchial epithelial cells (12). We previously reported that increased expression of SerpinB2 could promote airway T2 inflammation by regulating the expression of 15LO1, which play an essential role in the migration of eosinophils and promote airway NO production (14). Another *in vivo* study indicated that SerpinB2 could aggravate house dust mite induced airway inflammation and the T2 inflammatory response, suggesting that SerpinB2 might be a pivotal regulator in airway inflammation (24). Interestingly, we observed that exposure to exogenous SerpinB2 was associated with an increase in intracellular SerpinB2 levels. This phenomenon may reflect possible uptake of extracellular SerpinB2 by cells, or an indirect regulatory effect that enhances endogenous SerpinB2 expression. While the precise mechanism requires further investigation, these findings highlight a potential feedback process that could amplify the functional impact of SerpinB2 in airway epithelial responses. Combining previous studies with our results, we hypothesized that increased SerpinB2 in nasal mucosa could promote T2 inflammation and mucus hypersecretion, which aggravated the inflammation of nasal mucosa in CRSwNP.

We previously reported that SerpinB2 regulates IL-13 induced expression of the T2 “signature gene”, ALOX15 and downstream CCL26 and INOS. However, the specific function of SerpinB2 in AR is unclear. Pathological viscous mucus is the consequence of increased goblet cell number and therefore increased MUC5AC expression. Consistent with prior studies, we observed increased MUC5AC mRNA and protein expression after IL-13 treatment in nasal NECs. Pre-treatment with SerpinB2 siRNA was able to protect cultures when challenged with IL-13, with MUC5AC expression significantly decreased in the supernatants. In terms of MUC5B expression, prior literature is inconsistent, with some studies reporting increased MUC5B expression in AR, and others reporting no changes. In the current work, we observed no significant differences in MUC5B protein expression between the groups and as such, SerpinB2 siRNA treatment did not appear to cause any compensatory expression of MUC5B (data not shown).

POSTN is a well-characterized biomarker and crucial asthma regulator (23). POSTN promoted mucin hypersecretion and sustained eosinophilic inflammation, both of which were essential pathologies of asthma. (25, 26) POSTN was also a biomarker and a key player in tissue remodeling and fibrosis, which are important processes in bronchial asthma and nasal polyp. Previous studies suggest higher MUC5AC or lower MUC5B were correlated with T2 biomarkers. In addition, the higher MUC5AC were positively correlated with POSTN, which encodes periostin (T2 biomarker), and negatively correlated with Th1 cytokines. By contrast, the MUC5B were positively correlated with IL12A and negatively correlated with POSTN (27–29). In our previous study, *SerpinB2* gene were positive correlated with nasal epithelial cell *POSTN* in CRSwNP subjects. Our analysis strategy identified POSTN and SerpinB2 as core genes, which highlighted the significance of the two genes and supported the efficacy of our analyses. These finding suggests that the mechanism by which SerpinB2 regulated *POSTN* may be related to the above studies, as *POSTN* has been reported to be associated with abnormal airway epithelial EMT in asthma.

In this study, we demonstrated that SerpinB2 expression was markedly upregulated in NECs upon IL−13 stimulation and that its modulation impacted MUC5AC production through the STAT6 pathway. These findings suggest that SerpinB2 and STAT6 signaling participate actively in epithelial Th2 responses as previously reported (30, 31). Given that SerpinB2 is a regulator of the tPA/uPA system and inflammatory signaling (32), it may function not only as an intracellular checkpoint but also as a modulator of extracellular ligand−mediated crosstalk. Recent work has implicated STAT6−mediated ferroptosis and tPA−driven epithelial–fibroblast interactions in pulmonary fibrotic remodeling (33, 34). Accordingly, it is tempting to speculate that SerpinB2 could influence IL−13-induced MUC5AC expression in part via regulation of the tPA/uPA axis, thereby integrating extracellular protease activity with intracellular STAT6 signaling. Future studies will be required to test this hypothesis directly and define the specific ligand interactions involved.

Limitations of this study include the sample size is relatively small. Future study with larger sample size is warranted to validate the role of SerpinB2 in AR. The second is we classified AR into intermittent and persistent AR and we enrolled AR patients only allergic to dust mite. We did not classify allergic rhinitis by symptom severity (mild or moderate-severe), and we did not include patients allergic to allergens other than dust mites. It’s necessary to evaluate the role of SerpinB2 in different type of allergic rhinitis when more types of AR patients are available. Finally, the *in vivo* mouse model was not performed in this study. Future studies can use more advanced molecular biology techniques, such as gene knockout or overexpression mouse experiments to dissect SerpinB2 roles in AR.

In summary, our study firstly revealed a vital role of epithelial cell SerpinB2 in AR through regulating mucus hypersecretion. We identified higher SerpinB2 expression in AR epithelial cells, which was positively correlated with expression of MUC5AC. SerpinB2 further induced MUC5AC expression via STAT6 signaling activation. These findings provided mechanistic insights into SerpinB2 in mucus production and EMT remodeling of AR, and suggested the potential of SerpinB2 as a therapeutic target.

## Data Availability

The data presented in the study are deposited in the Gene Expression Omnibus repository, accession number GSE261706.
