# Supplementary material for: Elevated SerpinB2 regulates MUC5AC expression via STAT6 signaling in nasal epithelial cells in allergic rhinitis
Source: Front Immunol. 2025 Oct 10;16:1669777. doi: 10.3389/fimmu.2025.1669777 (PMC12549586; doi:10.3389/fimmu.2025.1669777)
Supplement: Supplementary file 1 [file DataSheet1.docx]

# Elevated SerpinB2 regulates MUC5AC expression via STAT6 signaling in nasal epithelial cells in allergic rhinitis

**Running Title: SerpinB2 regulates MUC5AC in allergic Rhinitis**

Jing Li ^a#^, Qianbo Cui ^a#^, Zhipeng Li ^b^, Ying Zhu ^b^, Jing Wang^c*^, Wei Chen ^a*^

^a^ Department of Otorhinolaryngology Head and Neck Surgery, The Central Hospital of Wuhan, Tongji Medical College, Huazhong University of Science and Technology, Hubei, China

^b^ Department of Otolaryngology Head and Neck Surgery, Shanghai Sixth People’s Hospital Affiliated to Shanghai Jiao Tong University School of Medicine, Shanghai, China

^c^ Department of Otorhinolaryngology Head and Neck Surgery, Tongji Hospital, Tongji Medical College, Huazhong University of Science and Technology, Hubei, China

**^#^Jing Li and Qianbo Cui contributed equally to this work.**

**Online Supplement**

**Methods**

*Reagents, Antibodies and Primers.*

BronchiaLife Epithelial Airway Medium Complete Kit was purchased from Lifeline Cell Technology (Carlsbad, CA, USA). Bronchial Epithelial Cell Basal medium (BEBM) and BEGM® SingleQuots were purchased from Lonza Company (Basel, Switzerland). Transwell cell culture plates were from Corning (Corning, NY, USA). SerpinB2 siRNA was from RiboBio (Guangzhou, China), and Lipofectamine RNAiMAX reagent was from Invitrogen (Carlsbad, CA, USA). Recombinant human IL-13 was purchased from R&D Systems (Minneapolis, MN, USA). SerpinB2 antibody were purchased from Proteintech (Minneapolis, MN, USA). GAPDH antibody was purchased from Proteintech (Littleton, CO, USA). Phospho-STAT6 antibody and total-STAT6 antibody were purchased from Cell Signaling Technology (Danvers, Massachusetts, USA). Real-Time PCR primers and probes were all purchased from EZBioscience. MUC5AC ELISA kit were purchased from R&D Systems (Minneapolis, MN, USA).

*Demographics*.

Fresh NECs were obtained from 15 HCs and 30 AR subjects. Due to the limited cell numbers, not all cells were used for all experiments. Fresh NEC analyses by Western blot were performed on 11 HC and 22 AR subjects (11 iAR and 11 pAR). mRNA analysis was performed on 10 HCs and 24 AR patients (12 iAR and 12 pAR). Immunostaining was performed on 8 HC and 16 NP participants (8 iAR and 8 pAR).

*Primary Nasal Epithelial Cell (NECs) Culture in Air–Liquid Interface (ALI).*

Primary human nasal epithelial cells (HNECs) were collected by epithelia scraping of the IT. Freshly harvested epithelial cells were placed directly into 10 mL of ice-cold PBS, centrifuged, washed, and resuspended in 1 mL of Lifeline BronchiaLife medium. A total of 1 × 10^5^ NECs were seeded into 60-mm tissue-culture dishes coated with rat tail type I collagen (BD, Franklin Lakes, NJ) containing 4 mL of BronchiaLife Basal Medium with BronchiaLife Life Factors Kit supplementation. Cells were cultured at 37℃ in a 5% CO_2_ environment. When epithelial cells reached around 90% confluence, they were dissociated with trypsin-EDTA and passed onto collagen (Advanced BioMatrix, Carlsbad, Calif)–coated polyester Transwell inserts of 12 mm in diameter (pore size, 0.4 mm) at 4 × 10^4^ cells/cm^2^ and continued in serum-free Dulbecco modified Eagle medium/Bronchial Epithelial Cell Basal medium supplemented with insulin (4 mg/mL), transferrin (5 mg/mL), hydrocortisone (0.5 mg/mL), epinephrine (0.5 mg/mL), bovine hypothalamus extract (52 mg/mL), gentamicin (50 mg/mL), amphotericin (50 ng/mL), bovine albumin (0.5 mg/mL), ethanolamine (80 nmol/L), MgCl2 (0.3 mmol/L), MgSO4 (0.4 mmol/L), CaCl2 (0.1 mmol/L), retinoic acid (30 ng/mL), and recombinant epidermal growth factor (0.5 ng/mL).

On reaching 100% confluency, cells were shifted to ALI culture by removing all but 50 μL of the apical medium. From day 0 of ALI, cells are stimulated with IL-13 (10 ng/mL) or SerpinB2 recombinant protein (R&D Systems) or medium alone added to the lower chamber every 48 hours up to 5 days. On day 5, cells were placed in 1mL TRIzol reagent for mRNA detection and in 300μL protein lysis buffer for WB analysis. Lower chamber supernatants were collected for MUC5AC ELISA tests.^1^

*DsiRNA transfection of primary NECs under ALI stage**.*

DsiRNA transfection was performed using Lipofectamine RNAiMAX reagent. Briefly, DsiRNA was pre-mixed with 3 μl per well Lipofectamine RNAiMAX transfection reagent in Opti-MEM medium for 20 minutes at room temperature before being pooled together with NECs suspension and seeded onto transwell. The final concentration of *SerpinB2* DsiRNA is 50nM. After 24 hours, the transfection mixture was removed and cells were switched to ALI culture. Cells were stimulated with or without IL-13 (10 ng/ml) under ALI culture for 5 days.^2^

*Western Blotting (WB).*

Fresh NECs from HC and AR patients and cultured cells were harvested in lysis buffer supplemented with Halt Protease Inhibitor Cocktail and Halt Phosphatase Inhibitor Cocktail (Thermo Fisher, Waltham, Mass). Volumes corresponding to 30μg protein were loaded and run on 4-12% SDS-PAGE Gel (EpiZyme Biotechnology). Proteins were transferred onto a nitrocellulose membrane (Pall Corporation) by using the eBlotTM L1 (GenScript). Membranes were blocked in 5% nonfat dry milk for at least 1 hour at room temperature and subsequently incubated overnight at 4℃ on a shaker with primary antibodies against SerpinB2 (1:1000 dilution) (16032-1-AP, Proteintech), GAPDH (1:10000 dilution) (Proteintech), Phospho-Stat6 (1:1000 dilution) (56554, Cell Signaling), and total-Stat6 (1:1000 dilution) (5397, Cell Signaling). After incubation with primary antibodies, the membranes were incubated with HRP-conjugated secondary antibodies for 1 hour at room temperature. The signals of the membrane were detected by chemiluminescence, and densitometric analysis was performed with ImageJ software.^3^

*qRT-PCR.*

Total RNA was extracted from tissues and cells from HCs and CRSwNP patients using TRIzol reagent (Invitrogen). A total of 1μg RNA was reverse-transcribed to cDNA using a Color Reverse Transcription Kit (A0010CGQ, EZBioscience). Quantitative PCR was performed with the SYBR Green Ⅰ method by using Color SYBR Green qPCR Master Mix (ROX2 plus) (A0012-R2, EZBioscience) with specific primers (Table E1). Amplification was as follows: 95 ℃ for 5 min, followed by 40 cycles of 95 ℃ for 10 sec, specific annealing temperature for 10 sec, and 60 ℃ for 30 sec. After PCR, a melting curve was constructed by increasing the temperature from 60 to 95 ℃ with a temperature transition rate of 0.11℃/s. Beta-glucuronidase (GUSB) was used as an internal control for normalization of gene expression. A normal inferior turbinate mucosal sample and a control cell culture sample were used as the calibrator for tissue samples and cell culture samples, respectively. An identical Ct was applied for each gene of interest. Relative mRNA expression was calculated by using the 2(-ΔCt) method.^4^

*Immunofluorescent staining and confocal microscopy.*

Tissue samples were ﬁxed in 4% formaldehyde solution and embedded in parafﬁn. Paraffin sections (4 μm) were prepared from paraffin-embedded human tissue samples.

Paraffin-embedded tissue sections were deparaffinized and hydrated. Then, antigen was retrieved with citrate antigen retrieval solution and blocked with 5% goat serum. The slides were incubated with primary antibodies against SerpinB2 (1:200 dilution) (16032-1-AP, Proteintech) and MUC5AC (1:100 dilution) (MA5-12178, Thermo Scientific) overnight in a wet chamber at 4℃ in the dark. The sections were then washed with PBS and incubated with Alexa Fluor 488- and Alexa Fluor 555- secondary antibodies for 1h at room temperature in the dark. After a final wash, cover slips were mounted onto the slides by using 4,6-diamidino-2-phenylindole for nuclear staining. Immunofluorescence images were acquired with a Zeiss confocal laser scanning microscope (LSM 780).^6^

*Bioinformatic analysis of the scRNA-seq dataset of AR subjects.*

Raw data of single cell RNA-sequencing (scRNA-seq) was downloaded from the Gene Expression Omnibus (GEO) database (accession number GSE261706). The gene-cell unique molecular identifier (UMI) count matrix was analyzed using R software (version 4.3.1) and the Seurat package (version 4.4.0). Low-quality cells were filtered out based on the following criteria: total UMI counts < 500, detected features < 250, and mitochondrial gene percentage > 35%. The filtered Seurat objects were then normalized, scaled, and integrated using Harmony for batch correction. Principal component analysis (PCA) was performed on highly variable genes for dimensionality reduction. For clustering and uniform manifold approximation and projection (UMAP) visualization, the first 15 principal components were selected based on visual inspection of the elbow plot, resulting in 18 clusters at a resolution of 0.5. Cell clusters were annotated using established marker genes: epithelial cells (*EPCAM, KRT8, KRT5*), mesenchymal cells (*COL1A1, ACTA2, PDGFRA*), and immune cells (*PTPRC, CD3E, MS4A1, HLA-DRA*). Small clusters expressing dual-lineage markers were identified as doublets and excluded from downstream analyses. Gene expression patterns were visualized using the FeaturePlot and VlnPlot functions within the Seurat package. Differential gene expression analysis between epithelial cells from control and AR groups was performed using the FindMarkers function.

**References**

1. Trudeau J, Hu H, Chibana K, Chu HW, Westcott JY, Wenzel SE. Selective downregulation of prostaglandin E2-related pathways by the Th2 cytokine IL-13. J Allergy Clin Immunol. 2006;117(6):1446-54.

2. Zhao J, Minami Y, Etling E, Coleman JM, Lauder SN, Tyrrell V, et al. Preferential Generation of 15-HETE-PE Induced by IL-13 Regulates Goblet Cell Differentiation in Human Airway Epithelial Cells. Am J Respir Cell Mol Biol. 2017;57(6):692-701.

3. Albano GD, Zhao J, Etling EB, Park SY, Hu H, Trudeau JB, et al. IL-13 desensitizes beta2-adrenergic receptors in human airway epithelial cells through a 15-lipoxygenase/G protein receptor kinase 2 mechanism. J Allergy Clin Immunol. 2015;135(5):1144-53 e1-9.

4. Xie M, Mustovich AT, Jiang Y, Trudeau JB, Ray A, Ray P, et al. IL-27 and type 2 immunity in asthmatic patients: association with severity, CXCL9, and signal transducer and activator of transcription signaling. J Allergy Clin Immunol. 2015;135(2):386-94.

5. Zhao J, Maskrey B, Balzar S, Chibana K, Mustovich A, Hu H, et al. Interleukin-13-induced MUC5AC is regulated by 15-lipoxygenase 1 pathway in human bronchial epithelial cells. Am J Respir Crit Care Med. 2009;179(9):782-90.

**Table S1. Primers used for quantitative RT-PCR analysis.**

| Gene name |  | Primer |
| --- | --- | --- |
| *SerpinB2* | Forword | CAGCACCGAAGACCAGATGG |
|  | Reverse | CCTGCAAAATCGCATCAGGATAA |
| *ALOX15* | Forword | GGGCAAGGAGACAGAACTCAA |
|  | Reverse | CAGCGGTAACAAGGGAACCT |
| *CCL26* | Forword | GGGAGTGACATATCCAAGACCTG |
|  | Reverse | CAGACTTTCTTGCCTCTTTTGGTA |
| *CCL24* | Forword | ACATCATCCCTACGGGCTCT |
|  | Reverse | CTTGGGGTCGCCACAGAAC |
| *POSTN* | Forword | CTCATAGTCGTATCAGGGGTCG |
|  | Reverse | ACACAGTCGTTTTCTGTCCAC |
| *MUC5AC* | Forword | ACGGTGCTTGACGACATCG |
|  | Reverse | GTATCCGGGCATGGAACCT |
| *MUC5B* | Forword | GCCTACGAGGACTTCAACGTC |
|  | Reverse | CCTTGATGACAACACGGGTGA |
| *GUSB* | Forword | GTCTGCGGCATTTTGTCGG |
|  | Reverse | CACACGATGGCATAGGAATGG |
